# Myxedema Secondary to Levothyroxine Malabsorption in Newly Diagnosed Celiac Disease

**DOI:** 10.7759/cureus.25491

**Published:** 2022-05-30

**Authors:** Brandon Wiggins, Kyle T Knight, Fady Banno, Mark Minaudo

**Affiliations:** 1 Internal Medicine, Ascension Genesys Hospital, Grand Blanc, USA; 2 Internal Medicine, Ascension Health, Grand Blanc, USA; 3 Gastroenterology and Hepatology, Ascension Health, Grand Blanc, USA

**Keywords:** hypothyroid myxedema coma, celiac disease (ced), celiac disease and thyroid, hashimoto's hypothyroidism, hashimoto’s thyroiditis

## Abstract

Celiac disease (CD) is an autoimmune disorder that predominantly affects the small intestine and is related to antibodies created against gluten when the substance is ingested. It is uncommon comorbidity in patients with Hashimoto's thyroiditis (HT). Myxedema is a severe form of hypothyroidism that is commonly related to new diagnoses, medication non-compliance, or malabsorption of thyroid supplementation that can have life-threatening associated conditions like heart failure and coma. In this article, we will describe a case of myxedema secondary to levothyroxine malabsorption in the setting of a newly diagnosed CD.

## Introduction

Celiac disease (CD) is an autoimmune disorder of the small intestine caused by sensitivity to dietary intake of the protein gluten. The prevalence of CD in the United States is approximately 0.75% of the population [1]. Of patients with confirmed CD, 95% are HLA-DQ2 positive and 5% are HLA-DQ8 positive [2]. An autoimmune response is initiated when the gliadin fraction of gluten binds via tissue transglutaminase to HLA-DQ2/DQ8 on antigen-presenting cells and is subsequently presented to T-cells and B-cells [3]. B-cell production of gliadin antibodies, tissue transglutaminase antibodies, and endomysium antibodies, as well as T-cell infiltration of the small intestinal mucosa, lead to characteristic blunting and atrophy of the intestinal villi [3]. Autoimmune damage, such as described, often leads to abdominal pain, diarrhea, as well as malabsorption [4]. Chronic malabsorption of vitamins, minerals, nutrients, and medications may lead to potentially severe metabolic disturbances [4].

Some 2%-5% of the population diagnosed with Hashimoto's thyroiditis (HT) have concurrent CD [5]. Chronic malabsorption of levothyroxine in the setting of HT with comorbid CD has been previously described in the literature [6]. These cases highlight the need for chronically higher doses of levothyroxine in such patients to achieve and maintain a euthyroid state. However, upon review of available medical literature via PubMed database, myxedema secondary to CD has only been reported once previously in 1964 by Kelley and Stewart [1, 4, 7].

Contrary to the name, myxedema coma usually presents with confusion and lethargy, but can progress to coma. Patients presenting with coma in myxedema coma usually are people that have had uncontrolled hypothyroidism for a longer period of time [8]. 

## Case presentation

A 55-year-old male with a past medical history of hypothyroidism and hyperlipidemia presented to the emergency department with the chief complaint of diarrhea. The patient stated that he had been experiencing non-bloody, mucoid diarrhea without any melena or hematochezia for years but it had acutely worsened over the past three months. He also had severe fatigue, confusion, and lethargy. The patient reported having a history of hypothyroidism but was unsure of the etiology.

On physical exam, the patient also had a distended abdomen and non-pitting edema in bilateral lower extremities that were cool to the touch. Vital signs were remarkable for blood pressure of 78/49 mmHg, and an oral temperature of 94 degrees Fahrenheit. His body mass index was 27.

**Figure 1:**
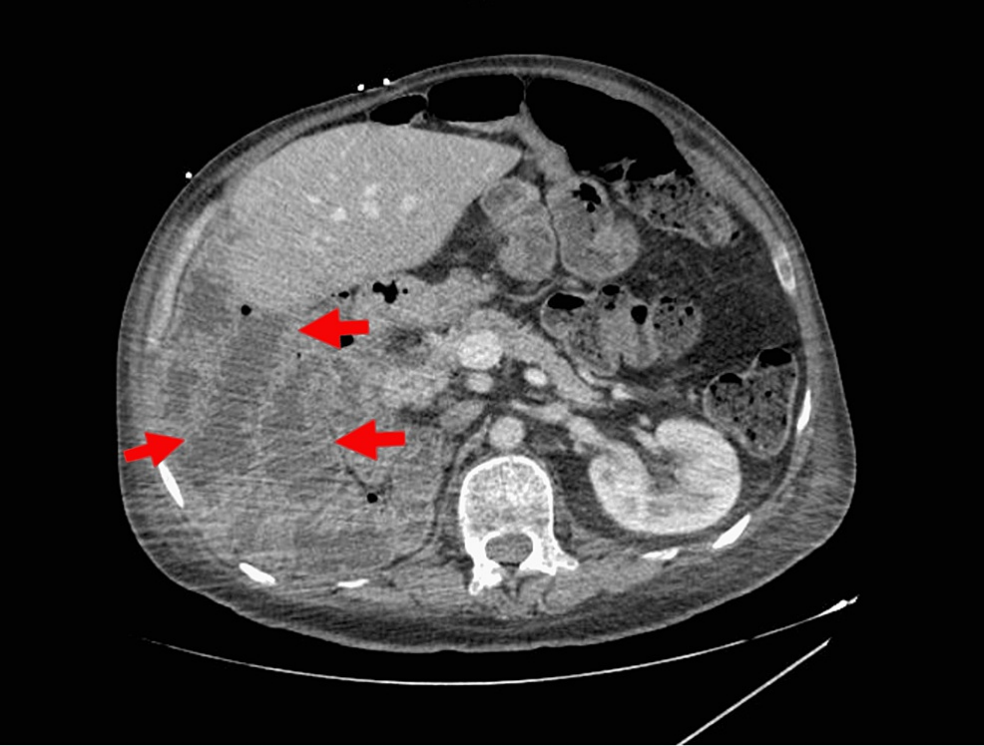
Transverse view CT of the abdomen. Red arrows demonstrating duodenal inflammation consistent with CD CD, celiac disease

Laboratory results on presentation were remarkable for thyroid stimulating hormone (TSH) of 79.6 uIU/mL with total thyroxine of 0.47 ng/dL and a total T3 of 53 ng/dL (Table 1). There was no baseline TSH, as the patient had never been hospitalized at our institution before. The patient had previously been compliant with oral 200 mcg levothyroxine at home daily over the last five years when he was originally diagnosed with HT. CT of the abdomen demonstrated inflammation of the small bowel (Figure 1). Ultrasound of bilateral lower extremities was negative for deep vein thrombosis (DVT). 

**Table 1:** Laboratory results on presentation.

Laboratory values	Measured	Normal range
White blood cell count	4.9 K/cm^2^	4.5–11 K/cm^2^
Hemoglobin	11.8 g/dL	11–16.2 g/dL
Hematocrit	36.9%	36%–46%
Platelet count	358 K/cm^2^	140–440 K/cm^2^
Serum sodium level	137 mmol/L	136–144 mmol/L
Serum potassium level	3.7 mmol/L	3.6–5.1 mmol/L
Serum chloride level	113 mmol/L	101–111 mmol/L
Total serum carbon dioxide	18 mmol/L	20–30 mmol/L
Serum blood urea nitrogen	12 mg/dL	8–26 mg/dL
Serum creatinine level	0.66 mg/dL	0.44–1.00 mg/dL
Anion gap	6 mmol/L	8–16 mmol/L
Serum glucose	88 mg/dL	70–99 mg/dL
Serum calcium level	7.1 mg/dL	8.4–10.2 mg/dL
Serum magnesium level	1.6 mg/dL	1.6–2.6 mg/dL
Serum phosphorous level	1.1 mg/dL	2.3–4.7 mg/dL
Total bilirubin	0.2 mg/dL	0.3–1 mg/dL
Serum albumin level	2.4 g/dL	3.5–5 g/dL
Aspartate aminotransferase	28 U/L	15–41 U/L
Alanine aminotransferase	30 U/L	14–54 U/L
Serum alkaline phosphatase	126 U/L	41–150 U/L
Thyroglobulin antibody	85.3 IU/mL	0–4 IU/mL
Thyroid peroxidase antibody	130.9 IU/mL	0–9 IU/mL
Transglutaminase antibody	>100 U/mL	0–3 U/mL
Endomysial antibody titer	1:80	< 1:10

On further investigation of myxedema, thyroglobulin antibody was 85.3 IU/mL and thyroid peroxidase enzyme was 130.9 IU/mL, consistent with HT. While investigating the differential diagnosis of chronic diarrhea, tissue transglutaminase antibody was over 100 U/mL, and endomysial immunoglobulin A (IgA) antibody titer was elevated at 1:80, consistent with a new diagnosis of CD. *Clostridium difficile* toxin and antigen, stool culture, parasite panel, and Shigatoxin were unremarkable. 

The patient was started on 180 mcg IV levothyroxine with the improvement of symptoms in one week. He was eventually stabilized and educated on the dietary restrictions involved with CD and started on 300 mcg of levothyroxine daily. On discharge, the patient's diarrhea and myxedema had resolved. He was referred to gastroenterology for management of newly diagnosed CD in the outpatient setting. 

## Discussion

This case describes a patient found to be in myxedema due to medication malabsorption in the setting of a newly diagnosed CD. To our knowledge myxedema secondary to medication malabsorption from undiagnosed CD has not been published before. This case is an important addition to the medical literature, as it highlights a unique etiology for myxedema, despite being compliant with medication. Future clinicians should consider malabsorption disorders, especially CD, when patients are compliant with thyroid supplementation but still have uncontrolled hypothyroidism and the complications that may be associated, such as heart failure exacerbations. 

Malabsorption refers to the impaired transportation of nutrients, carbohydrates, fats, micronutrients, or medications across the apical membrane of enterocytes and into the bloodstream [8]. HT, the most common form of hypothyroidism, requires daily lifelong treatment with oral synthetic thyroid hormones [9]. Previous studies have demonstrated a greater requirement of levothyroxine to maintain a euthyroid state in patients with hypothyroidism and concurrent untreated CD, likely related to medication malabsorption [1, 9-10].

Guidelines suggest that patients with newly diagnosed hypothyroidism should be started on a levothyroxine dose of approximately 1.6 mcg/kg of body weight per day [11]. There are no current universal screening recommendations for CD in patients with HT. There are also no dosing recommendations for patients that have concurrent CD and HT. Studies carried out at the University of Vermont demonstrated an increasing prevalence of CD as levothyroxine dosage increased (5.6% at doses >125 mcg/daily and 12.5% at >200 mcg/daily) or if patients required greater than 1.5 mcg/kg/day for maintenance therapy [1, 4]. Authors of these studies argue for serologic testing for CD in patients requiring a normal body mass index (BMI) and >125 mcg/daily of levothyroxine [1, 4]. 

Until empirical and/or universal screening recommendations for CD in HT patients are implemented, clinicians should be aware of the small but significant comorbidity rate of CD and HT, and test for CD in cases of high clinical suspicion. Increases in levothyroxine requirements to maintain a euthyroid state should be another cause for concern regarding the possible presence of new-onset CD. As demonstrated in University of Vermont studies, appropriate treatment of CD with a gluten-free diet has been shown to reduce the amount of levothyroxine required to achieve a euthyroid state [1, 4]. HT patients with comorbid CD should, therefore, be recommended a gluten-free diet in addition to appropriate adjustment of thyroid treatment to achieve and maintain a euthyroid state.

## Conclusions

Myxedema coma in the setting of newly diagnosed CD is a previously undescribed phenomenon and we would like to alert clinicians to this association. Contrary to the name, myxedema coma can present as confusion and lethargy as opposed to coma, as seen in this case, which is an important point that needs to be highlighted as well. Myxedema coma also can commonly present with bradycardia, hypothermia, hypotension, and classic edema of the lower extremities. Management of concurrent CD and HT is difficult because levothyroxine malabsorption is dependent on the severity of CD, which is widely variable. This is an area of interest for the gastrointestinal and endocrinology research communities to focus on in the future.
